# Supplementary figures and images for: Assessment of decadal changes in coastal nitrogen sources in NW Spain with stable isotopes in macroalgae and mussels
Source: PLoS One. 2025 Jul 1;20(7):e0327159. doi: 10.1371/journal.pone.0327159 (PMC12212548; doi:10.1371/journal.pone.0327159)

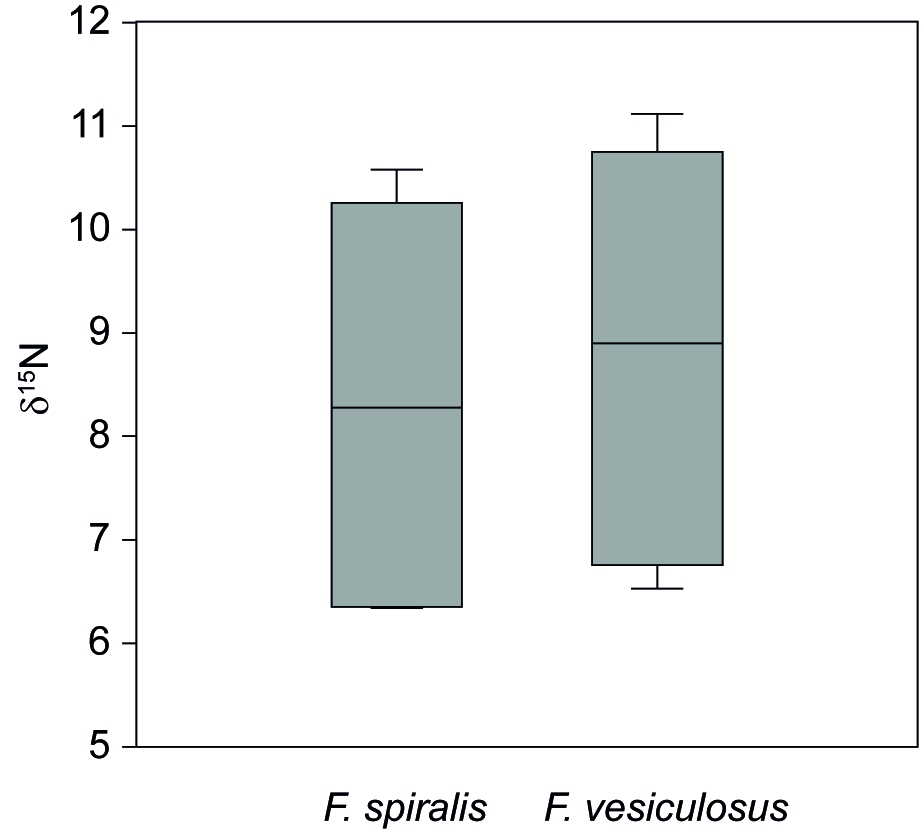

Supplement: S1 Fig — Annual series of monthly averaged values for the upwelling index (UI, m3 s-1 km-1) for the three areas considered (see Fig 1). The continuous line indicates the center of gravity (weighted average of UI) of the main upwelling peak each year. (ZIP) [file pone.0327159.s001.zip › FigS3.tif]

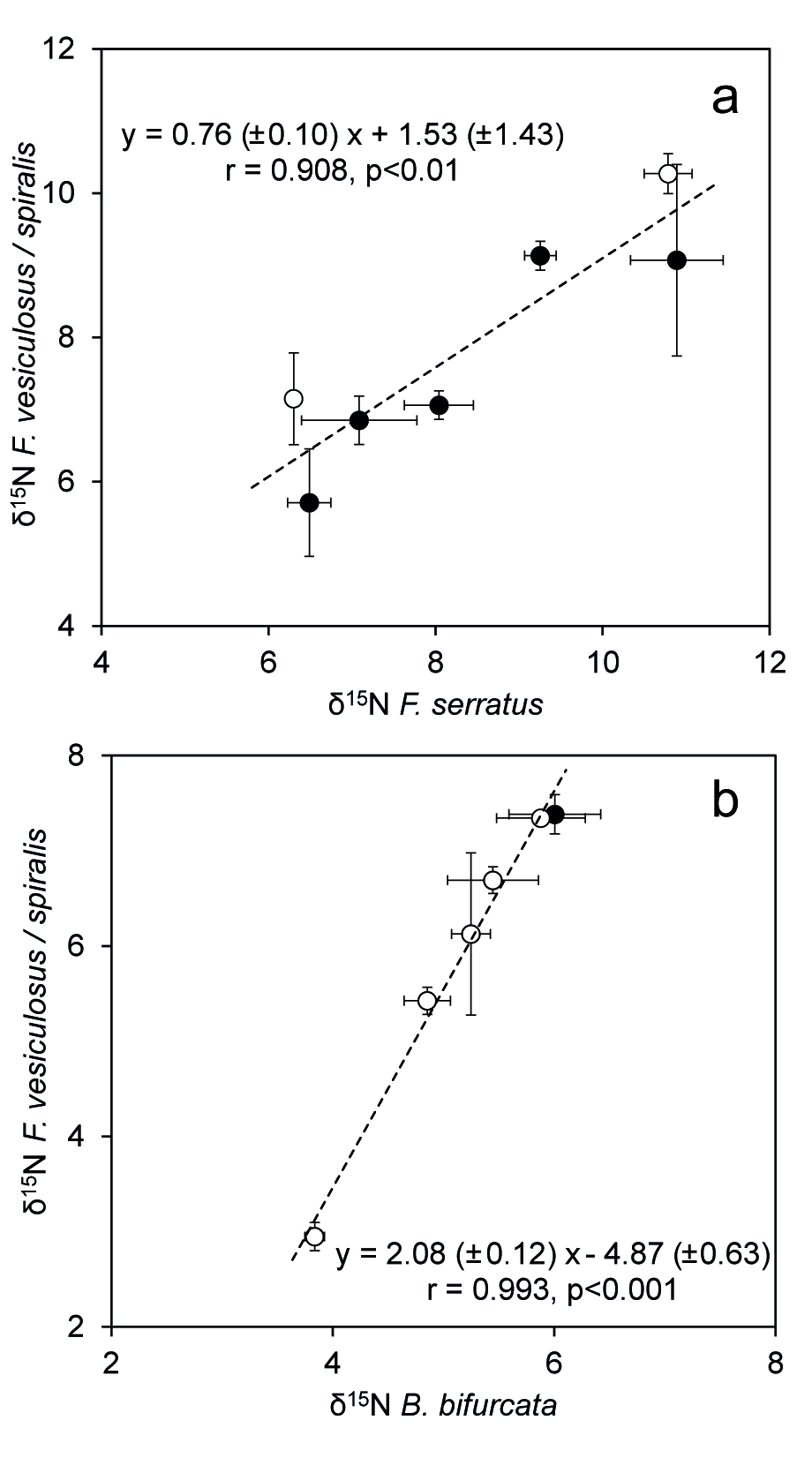

Supplement: S1 Fig — Annual series of monthly averaged values for the upwelling index (UI, m3 s-1 km-1) for the three areas considered (see Fig 1). The continuous line indicates the center of gravity (weighted average of UI) of the main upwelling peak each year. (ZIP) [file pone.0327159.s001.zip › FigS4.tif]

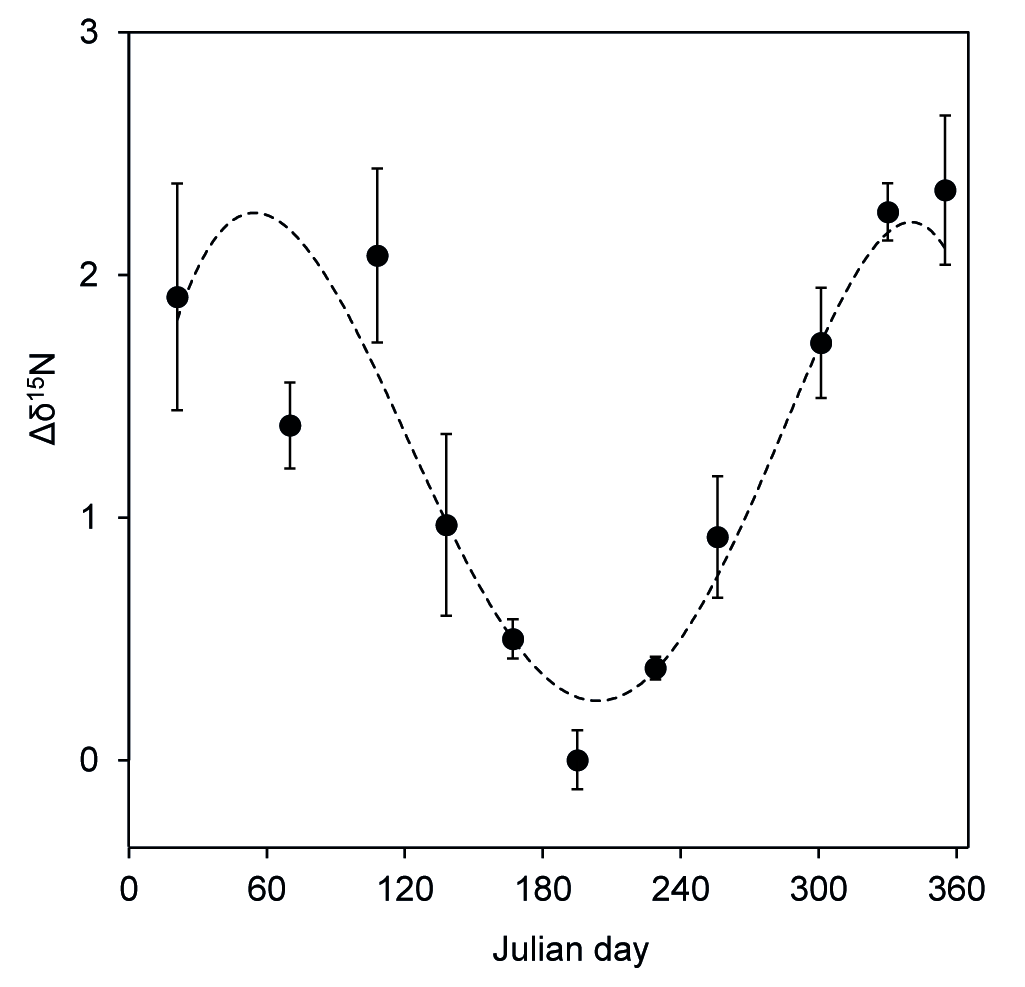

Supplement: S1 Fig — Annual series of monthly averaged values for the upwelling index (UI, m3 s-1 km-1) for the three areas considered (see Fig 1). The continuous line indicates the center of gravity (weighted average of UI) of the main upwelling peak each year. (ZIP) [file pone.0327159.s001.zip › FigS5.tif]

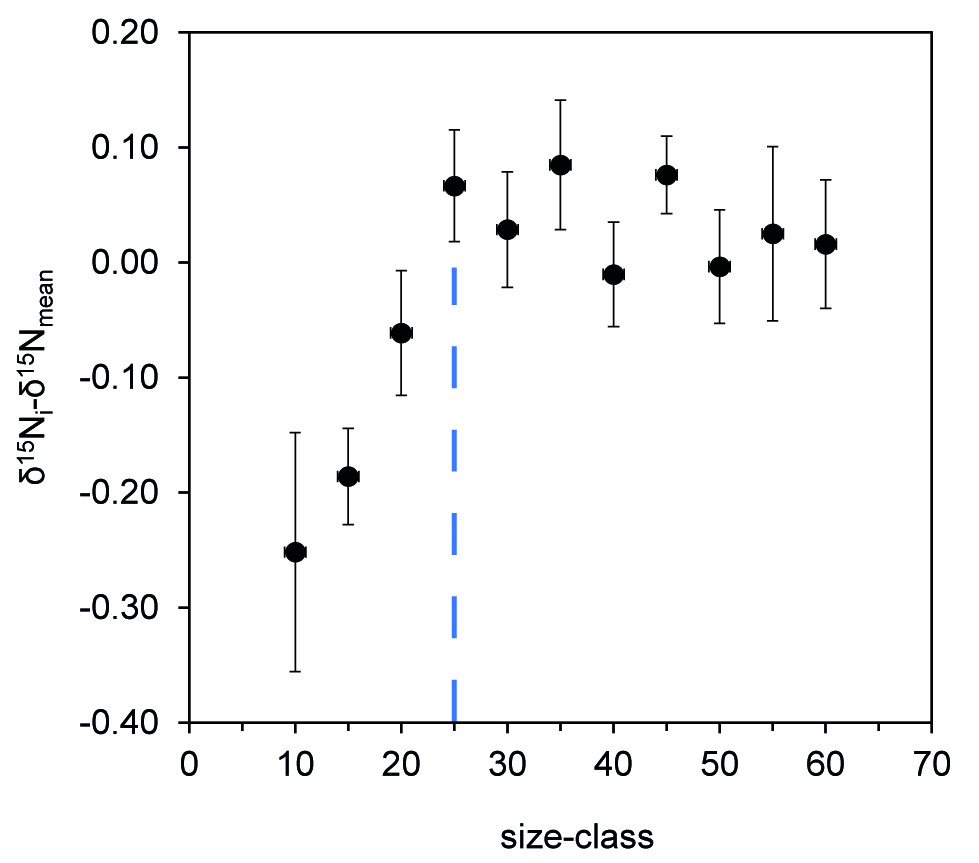

Supplement: S1 Fig — Annual series of monthly averaged values for the upwelling index (UI, m3 s-1 km-1) for the three areas considered (see Fig 1). The continuous line indicates the center of gravity (weighted average of UI) of the main upwelling peak each year. (ZIP) [file pone.0327159.s001.zip › FigS6.tif]

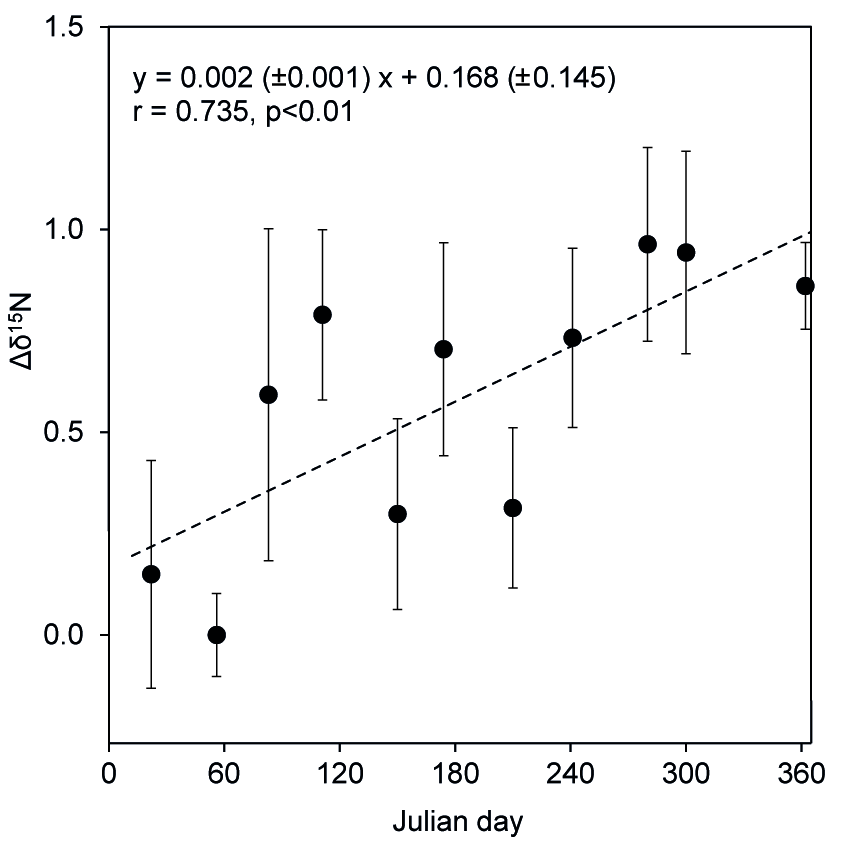

Supplement: S1 Fig — Annual series of monthly averaged values for the upwelling index (UI, m3 s-1 km-1) for the three areas considered (see Fig 1). The continuous line indicates the center of gravity (weighted average of UI) of the main upwelling peak each year. (ZIP) [file pone.0327159.s001.zip › FigS7.tif]

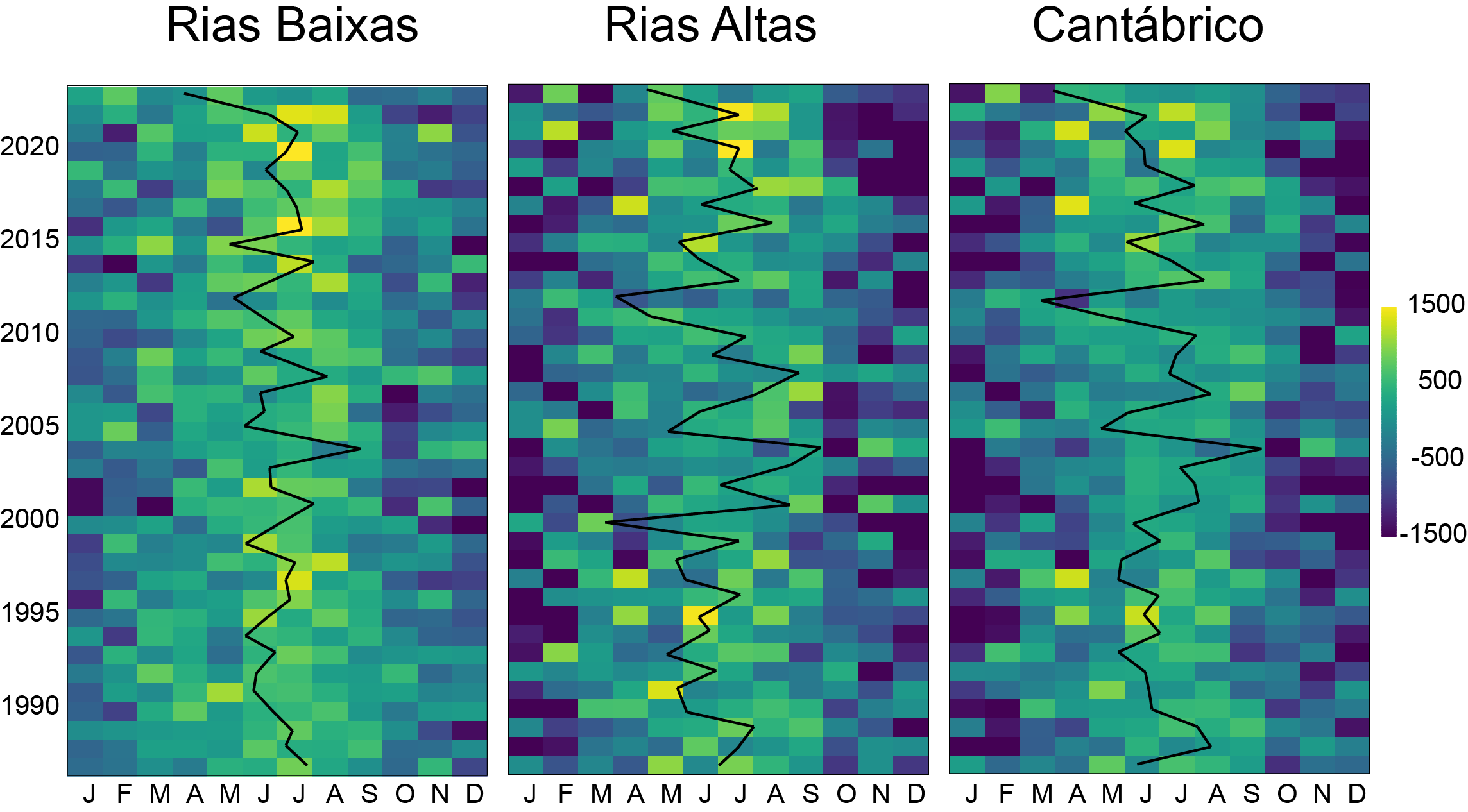

Supplement: S1 Fig — Annual series of monthly averaged values for the upwelling index (UI, m3 s-1 km-1) for the three areas considered (see Fig 1). The continuous line indicates the center of gravity (weighted average of UI) of the main upwelling peak each year. (ZIP) [file pone.0327159.s001.zip › FigS1.tif]

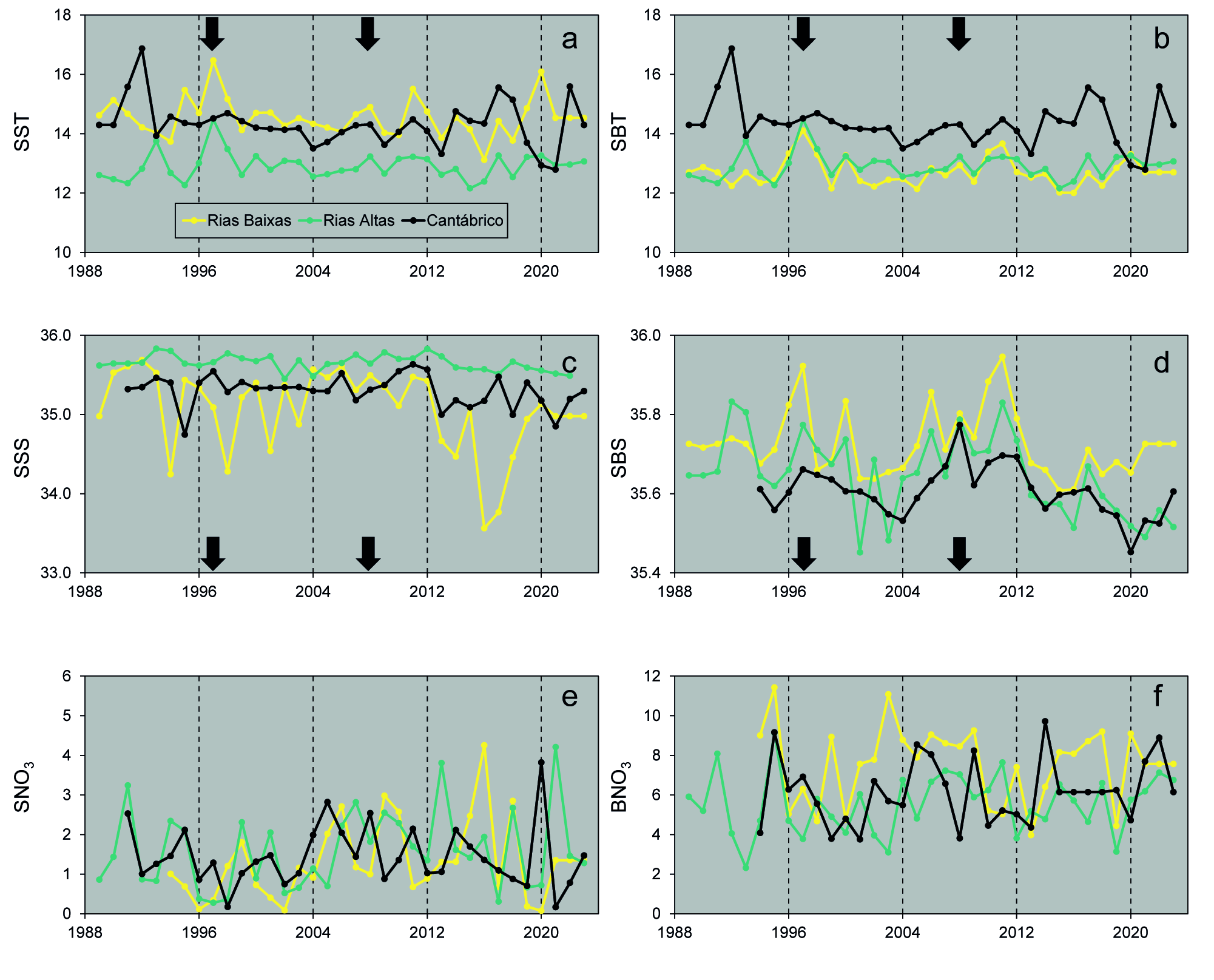

Supplement: S1 Fig — Annual series of monthly averaged values for the upwelling index (UI, m3 s-1 km-1) for the three areas considered (see Fig 1). The continuous line indicates the center of gravity (weighted average of UI) of the main upwelling peak each year. (ZIP) [file pone.0327159.s001.zip › FigS2.tif]
