# Supplementary material for: Assessment of decadal changes in coastal nitrogen sources in NW Spain with stable isotopes in macroalgae and mussels
Source: PLoS One. 2025 Jul 1;20(7):e0327159. doi: 10.1371/journal.pone.0327159 (PMC12212548; doi:10.1371/journal.pone.0327159)
Supplement: Attachment — Submitted filename: response_letter.docx [file pone.0327159.s009.docx]

Academic Editor

PLOS ONE

Ref: PONE-D-24-58553. Assessment of decadal changes in coastal nitrogen sources in NW Spain with stable isotopes in macroalgae and mussels. PLOS ONE

Dear Editor:

We sincerely appreciate your time and the effort of the reviewers in evaluating the manuscript. We have carefully considered all reviews and editorial comments and have revised the manuscript accordingly. We believe this revision has significantly enhanced the text, making it more focused and targeted. Below, I provide point by point responses to the issues and comments raised by the editor and reviewers (original comments in regular type and responses in **boldface**). We also provide additional references supporting our argument at the end of this letter.

Please note my current e-mail address for further correspondence ([antonio.bode@ieo.csic.es](mailto:antonio.bode@ieo.csic.es)), as the evaluation report was sent to a wrong address.

Sincerely,

Antonio Bode, on behalf of all the authors

Dear Dr. Bode,

Thank you for submitting your manuscript to PLOS ONE. After careful consideration, we feel that it has merit but does not fully meet PLOS ONE’s publication criteria as it currently stands. Therefore, we invite you to submit a revised version of the manuscript that addresses the points raised during the review process.

Please submit your revised manuscript by May 10 2025 11:59PM. If you will need more time than this to complete your revisions, please reply to this message or contact the journal office at [plosone@plos.org](mailto:plosone@plos.org). When you're ready to submit your revision, log on to https://www.editorialmanager.com/pone/ and select the 'Submissions Needing Revision' folder to locate your manuscript file.

Please include the following items when submitting your revised manuscript:

- A rebuttal letter that responds to each point raised by the academic editor and reviewer(s). You should upload this letter as a separate file labeled 'Response to Reviewers'.
- A marked-up copy of your manuscript that highlights changes made to the original version. You should upload this as a separate file labeled 'Revised Manuscript with Track Changes'.
- An unmarked version of your revised paper without tracked changes. You should upload this as a separate file labeled 'Manuscript'.

If you would like to make changes to your financial disclosure, please include your updated statement in your cover letter. Guidelines for resubmitting your figure files are available below the reviewer comments at the end of this letter.

If applicable, we recommend that you deposit your laboratory protocols in protocols.io to enhance the reproducibility of your results. Protocols.io assigns your protocol its own identifier (DOI) so that it can be cited independently in the future. For instructions see: <https://journals.plos.org/plosone/s/submission-guidelines#loc-laboratory-protocols>. Additionally, PLOS ONE offers an option for publishing peer-reviewed Lab Protocol articles, which describe protocols hosted on protocols.io. Read more information on sharing protocols at <https://plos.org/protocols?utm_medium=editorial-email&utm_source=authorletters&utm_campaign=protocols>.

We look forward to receiving your revised manuscript.

Kind regards,

Rajdeep Roy

Academic Editor

PLOS ONE

**Journal Requirements:**

1. When submitting your revision, we need you to address these additional requirements.

Please ensure that your manuscript meets PLOS ONE's style requirements, including those for file naming. The PLOS ONE style templates can be found at

<https://journals.plos.org/plosone/s/file?id=wjVg/PLOSOne_formatting_sample_main_body.pdf>	and

<https://journals.plos.org/plosone/s/file?id=ba62/PLOSOne_formatting_sample_title_authors_affiliations.pdf>

**The new version conforms with the journal style following the templates**

2. In your Methods section, please provide additional information regarding the permits you obtained for the work. Please ensure you have included the full name of the authority that approved the field site access and, if no permits were required, a brief statement explaining why.

**We have detailed in the methods section the sampling permits obtained:**

**“The collection of intertidal specimens was done under permits granted by Xunta de Galicia, Spain (Consellería de Medio Ambiente, permit Environmental Specimen Bank project, and Consellería do Mar, permit 22062010 ANILE project)”**

3. Thank you for stating in your Funding Statement:

This research was supported by MCIN/AEI/10.13039/501100011033 (Spain) through project QLOCKS (PID2020-115620RB-100), and additional funds by the Consellería de Medio Ambiente (Galicia, Spain) through the 3rd phase of the Environmental Specimen Bank (2000–2004), the Competitive Reference Group GRC GI-1252/GPC2020-23 (ED431C 2020/19), co-funded by the Xunta de Galicia (Spain) and the European Regional Development Fund (ERDF, EU), and postdoctoral research grants Juan de la Cierva-Incorporación to I.G.V. (IJC2019-040554-I), Juan de la Cierva-Formación to R.G.S. (FJC2019-040921-I), and María Zambrano Programme of the Spanish Ministry of Universities to Z.V. R.G.S also received funds from the European Union NextGenerationEU/PRTR programmes and is currently supported by the Horizon Europe research and innovation programme under a Marie Skłodowska-Curie Postdoctoral Fellowship 2023 (101150001-PelCon).

Please provide an amended statement that declares *all* the funding or sources of support (whether external or internal to your organization) received during this study, as detailed online in our guide for authors at http://journals.plos.org/plosone/s/submit-now.  Please also include the statement “There was no additional external funding received for this study.” in your updated Funding Statement.

Please include your amended Funding Statement within your cover letter. We will change the online submission form on your behalf.

**We have amended the funding paragraph stating all the funding sources and the names of beneficiaries when relevant:**

**“This research was supported by MCIN/AEI/10.13039/501100011033 (Spain) through projects ANILE (CTM2009-08396 and CTM2010-08804-E) and QLOCKS (PID2020-115620RB-100), by IEO time-series project RADIALES, and additional funds by the Consellería de Medio Ambiente (Galicia, Spain) through the 3rd phase of the Environmental Specimen Bank (2000–2004), the Competitive Reference Group GRC GI-1252/GPC2020-23 (ED431C 2020/19), co-funded by the Xunta de Galicia (Spain) and the European Regional Development Fund (ERDF, EU), and postdoctoral research grants Juan de la Cierva-Incorporación to I.G.V. (IJC2019-040554-I), Juan de la Cierva-Formación to R.G.S. (FJC2019-040921-I), and María Zambrano Programme of the Spanish Ministry of Universities to Z.V. R.G.S also received funds from the European Union NextGenerationEU/PRTR programmes and is currently supported by the Horizon Europe research and innovation programme under a Marie Skłodowska-Curie Postdoctoral Fellowship 2023 (101150001-PelCon). There was no additional external funding received for this study.”**

4. We noted in your submission details that a portion of your manuscript may have been presented or published elsewhere. Small subsets of the data on nitrogen stable isotopes in macroalgae were published in references 47, 49, 50, 56 and 57, as indicated in the manuscript. These data were used for different purposes of the objective of the present study. Please clarify whether this [conference proceeding or publication] was peer-reviewed and formally published. If this work was previously peer-reviewed and published, in the cover letter please provide the reason that this work does not constitute dual publication and should be included in the current manuscript.

**The isotopic data from previous studies was employed in peer-reviewed research articles with purposes different from the objective of this study. For instance, Viana et al. [46] used *Fucus* stable isotopes to detect coastal eutrophication in sheltered areas of Galicia between 1990 and 2007. In turn, Viana and Bode [45, 48] and Bode et al. [54] focused on the detection of the influence of anthropogenic vs. marine nitrogen sources at local or regional scales, respectively, but these were pilot studies considering only or two years of data. Finally, Garcia-Seoane et al. [55] used a small subset of macroalgal data to apply new isotope mixing models to food webs.**

**In this manuscript we added ca. 10% and 6% to the total isotopic data of algae and mussels, respectively, from samples collected in 2023. All these raw data were not published before nor used with the objective of examining nitrogen sources over such a long time period as in the present manuscript. All the isotopic data used is now available through the PANGAEA repository [61, 62]**

5. When completing the data availability statement of the submission form, you indicated that you will make your data available on acceptance. We strongly recommend all authors decide on a data sharing plan before acceptance, as the process can be lengthy and hold up publication timelines. Please note that, though access restrictions are acceptable now, your entire data will need to be made freely accessible if your manuscript is accepted for publication. This policy applies to all data except where public deposition would breach compliance with the protocol approved by your research ethics board. If you are unable to adhere to our open data policy, please kindly revise your statement to explain your reasoning and we will seek the editor's input on an exemption. Please be assured that, once you have provided your new statement, the assessment of your exemption will not hold up the peer review process.

**Now all the data used in this manuscript is available through international repositories. The Data availability section has been modified as follows:**

**“Upwelling index values are available at** [**http://www.indicedeafloramiento.ieo.es**](http://www.indicedeafloramiento.ieo.es)**. Hydrographic data are available at** [**https://doi.org/10.1594/PANGAEA.919087**](https://doi.org/10.1594/PANGAEA.919087) **and at** [**https://cdi.seadatanet.org/csr/**](https://cdi.seadatanet.org/csr/) **(project RADIALES). Stable isotope data are available at** [**https://doi.org/10.1594/PANGAEA.974186**](https://doi.org/10.1594/PANGAEA.974186) **and**

[**https://doi.org/10.1594/PANGAEA.974299**](https://doi.org/10.1594/PANGAEA.974299)**”**

6. We note that you have referenced “Bode A, García-Seoane R, Varela Z, Viana IG” which has currently not yet been accepted for publication. Please remove this from your References and amend this to state in the body of your manuscript: (“Bode A, García-Seoane R, Varela Z, Viana IG”. [Submitted]) as detailed online in our guide for authors

<http://journals.plos.org/plosone/s/submission-guidelines#loc-reference-style>

**All isotopic data used in the manuscript are now published. We now provide full references with DOI: references [61, 62]**

7. We note that Figure 1 in your submission contain map images which may be copyrighted. All PLOS content is published under the Creative Commons Attribution License (CC BY 4.0), which means that the manuscript, images, and Supporting Information files will be freely available online, and any third party is permitted to access, download, copy, distribute, and use these materials in any way, even commercially, with proper attribution. For these reasons, we cannot publish previously copyrighted maps or satellite images created using proprietary data, such as Google software (Google Maps, Street View, and Earth). For more information, see our copyright guidelines: http://journals.plos.org/plosone/s/licenses-and-copyright.

We require you to either present written permission from the copyright holder to publish these figures specifically under the CC BY 4.0 license, or remove the figures from your submission:

a. You may seek permission from the original copyright holder of Figure 1 to publish the content specifically under the CC BY 4.0 license.

We recommend that you contact the original copyright holder with the Content Permission Form (http://journals.plos.org/plosone/s/file?id=7c09/content-permission-form.pdf) and the following text:

“I request permission for the open-access journal PLOS ONE to publish XXX under the Creative Commons Attribution License (CCAL) CC BY 4.0 (http://creativecommons.org/licenses/by/4.0/). Please be aware that this license allows unrestricted use and distribution, even commercially, by third parties. Please reply and provide explicit written permission to publish XXX under a CC BY license and complete the attached form.”

Please upload the completed Content Permission Form or other proof of granted permissions as an "Other" file with your submission.

In the figure caption of the copyrighted figure, please include the following text: “Reprinted from [ref] under a CC BY license, with permission from [name of publisher], original copyright [original copyright year].”

b. If you are unable to obtain permission from the original copyright holder to publish these figures under the CC BY 4.0 license or if the copyright holder’s requirements are incompatible with the CC BY 4.0 license, please either i) remove the figure or ii) supply a replacement figure that complies with the CC BY 4.0 license. Please check copyright information on all replacement figures and update the figure caption with source information. If applicable, please specify in the figure caption text when a figure is similar but not identical to the original image and is therefore for illustrative purposes only.

The following resources for replacing copyrighted map figures may be helpful:

USGS National Map Viewer (public domain): http://viewer.nationalmap.gov/viewer/

The Gateway to Astronaut Photography of Earth (public domain): http://eol.jsc.nasa.gov/sseop/clickmap/

Maps at the CIA (public domain): https://www.cia.gov/library/publications/the-world-factbook/index.html and https://www.cia.gov/library/publications/cia-maps-publications/index.html

NASA Earth Observatory (public domain): http://earthobservatory.nasa.gov/

Landsat: http://landsat.visibleearth.nasa.gov/

USGS EROS (Earth Resources Observatory and Science (EROS) Center) (public domain): http://eros.usgs.gov/#

Natural Earth (public domain): <http://www.naturalearthdata.com/>

**Fig. 1 was redrawn using Ocean Data View software with permission to reproduce maps and graphs in scientific publications. We have included in the caption the reference, as required by the software providers (Schlitzer, R., Ocean Data View, https://odv.awi.de, 2025).**

**Below we copy the permit from the software provider as stated in https://odv.awi.de:**

**“Ocean Data View is allowed to be used free of charge for non-commercial, non-military research and teaching purposes only. If you use the software for your scientific work, please reference Ocean Data View in your publications as follows: Schlitzer, Reiner, Ocean Data View, https://odv.awi.de, 2025.”**

**Additional Editor Comments:**

Comments to the authors

Since as a handling editor I had difficult times in getting your manuscript reviewed I took the liberty to do it my self being from the similar field. In addition to the comments received I have some more observations which I mention below. The authors are encouraged to go through.
The authors try to understand the relationships between the nitrogen uptake through N15 isotope (during the upwelling period) on macroalgae and the mussel. The data is collected over the years during the upwelling period.

**We thank the editor for understanding the main results and the implications of our findings.**

My first question is both the experimental species are located in near shore/intertidal. One is a filter feeder and other I guess is attached to the substrata in very shallow waters. Whether the sampling location is affected by the upwelled waters.? How does authors prove this? This is not reflected by the salinity or temperature although some figures are given? The location is right next to shore so whether the hypothesis that upwelled might influence nitrate uptake is flawed? The authors need to justify. Why not change the orientation of the paper to “N15 isotope assimilation by intertidal species”?. Upwelling could be discussed as a paragraph in the discussion. Probably that’s the reason there is no change observed!

**Thanks for pointing out this issue. Nutrient inputs by coastal upwelling strongly influence marine productivity at large spatial scales, from offshore waters to intertidal communities. The influence of upwelling nitrogen has been successfully tracked in intertidal organisms using stable isotopes in several major upwelling regions as South Africa [30], California [31], or North Chile [32].**

**Upwelling is also the major source of new nitrogen for marine ecosystems in the study region, and is considered the main responsible of their high fish and shellfish production (Figueiras et al. 2002, Arístegui et al. 2006, [42]). There is ample evidence that most of the nitrogen required for phytoplankton primary production in the western region (Galicia) is provided by upwelling particularly in offshore waters (e.g. [12, 13]). Coastal upwelling during summer provide environmental conditions similar to those at more northern latitudes thus allowing the presence of cold-temperate canopy and creating sharp biogeographic boundaries in this region (Casado-Amezúa et al. 2019, [53]). Our previous studies in this region tracked upwelling nitrogen in intertidal macroalgae [45, 48] and fauna (Bode et al., 2011, [54].**

**We added a clarification on the influence of upwelling in intertidal species in the Introduction: “In turn, stable isotopes in intertidal macrophytes tracked the geographic variability in upwelling along the coast [45] but pointed out to a large prevalence of anthropogenic nutrients, at least in sheltered areas inside the rías [45 – 49].”**

**Based on these evidences, we reconstructed long time series of natural abundance of stable nitrogen isotopes in macroalgae and mussels to trace nitrogen sources at the northern boundary of the Canary Current upwelling system (NW Spain) between 1989 and 2023. Specifically, we examined the decadal variability of upwelling strength on nitrate supply and its effects on nitrogen stable isotope composition on the base of the marine food web.**

**We appreciated the suggestion of a change in the orientation of the paper but find that the “N15 isotope assimilation by intertidal species” is already sufficiently demonstrated by previous studies (e.g. [30, 31, 32, 45-48, 54], as noted above. Therefore, we prefer maintaining the objective of the study focused on the variability of upwelling nitrogen supply.**

Mussel responds strongly to calcium carbonate saturation and temperature fluctuation! The authors are encouraged to make a plot of showing the spatial extent of upwelling for any particular year to substantiate their scientific hypothesis.

**We also acknowledge that mussel physiology may be strongly affected by external factors as temperature and calcium carbonate saturation, thus potentially also affecting feeding and nitrogen metabolism. However, we tried to minimize these effects by constraining the time of sampling and the size of individuals analyzed (Figs S6 and S7).**

Also encouraged to plot the actual nitrate values from the location during monsoon and non-monsoon to show if any influence?

**We understand that the editor refers to upwelling vs. non-upwelling periods instead of ‘monsoon and non-monsoon’. Actual nitrate values for the beginning of the upwelling period at sampling sites representative of the different subregions are reported in Fig. S2 because in this paper we focus on the influence of new nitrogen provided by the upwelling. Previous studies with zooplankton in this region [43, 44] demonstrated that the isotopic signal of upwelling nitrogen was evident at the beginning of the upwelling season (March-June) while it was subsequently diluted by the use of regenerated nitrogen during late summer and autumn. Detailed examples of the variability in upwelling and nutrient concentrations at seasonal or daily time scales can be found in previous publications (e.g. [5, 22].**

A table showing a comparison of N15 assimilation by the (Mytilus galloprovincialis) from different other geographical area and for the macro algae can be added along with some discussion.
With the above suggestion the authors are encouraged to revise the present manuscript….

**While nitrogen isotopes were used as a tracers of upwelling in mussels from other regions, as South Africa [30], California [31], or North Chile [32], the actual δ^15^N values are only useful for regional comparison. Because of the different contribution of nitrification and denitrification processes to the formation of nitrate in each region there are characteristic reference δ^15^N values (or ‘baseline’ δ^15^N e.g. [31]). Therefore, the comparison of δ^15^N values of mussels from very different regions is not useful. The same occurs with the macroalgae δ^15^N. However, the comparison of δ^15^N values from different locations within the same region is a valuable tool to detect the biographical influence of upwelling [31-33, 45].**

[Note: HTML markup is below. Please do not edit.]

Reviewers' comments:

Reviewer's Responses to Questions

**Comments to the Author**

1. Is the manuscript technically sound, and do the data support the conclusions?

The manuscript must describe a technically sound piece of scientific research with data that supports the conclusions. Experiments must have been conducted rigorously, with appropriate controls, replication, and sample sizes. The conclusions must be drawn appropriately based on the data presented.

Reviewer #1: Yes

**We thank the reviewer for understanding the main results of our article.**

2. Has the statistical analysis been performed appropriately and rigorously?

Reviewer #1: I Don't Know

**We note that the statistical methods used in this study were previously applied to subsets of the same data, either considering the upwelling index [22] or stable isotopes [45, 46, 48, 54]**

3. Have the authors made all data underlying the findings in their manuscript fully available?

The [PLOS Data policy](http://www.plosone.org/static/policies.action#sharing) requires authors to make all data underlying the findings described in their manuscript fully available without restriction, with rare exception (please refer to the Data Availability Statement in the manuscript PDF file). The data should be provided as part of the manuscript or its supporting information, or deposited to a public repository. For example, in addition to summary statistics, the data points behind means, medians and variance measures should be available. If there are restrictions on publicly sharing data—e.g. participant privacy or use of data from a third party—those must be specified.

Reviewer #1: No

**Now all the data used in this manuscript is available through international repositories. The Data availability section has been modified as follows:**

**“Upwelling index values are available at** [**http://www.indicedeafloramiento.ieo.es**](http://www.indicedeafloramiento.ieo.es)**. Hydrographic data are available at** [**https://doi.org/10.1594/PANGAEA.919087**](https://doi.org/10.1594/PANGAEA.919087) **and at** [**https://cdi.seadatanet.org/csr/**](https://cdi.seadatanet.org/csr/) **(project RADIALES). Stable isotope data are available at** [**https://doi.org/10.1594/PANGAEA.974186**](https://doi.org/10.1594/PANGAEA.974186) **and** [**https://doi.org/10.1594/PANGAEA.974299**](https://doi.org/10.1594/PANGAEA.974299)**”**

4. Is the manuscript presented in an intelligible fashion and written in standard English?

PLOS ONE does not copyedit accepted manuscripts, so the language in submitted articles must be clear, correct, and unambiguous. Any typographical or grammatical errors should be corrected at revision, so please note any specific errors here.

Reviewer #1: Yes

5. Review Comments to the Author

Please use the space provided to explain your answers to the questions above. You may also include additional comments for the author, including concerns about dual publication, research ethics, or publication ethics. (Please upload your review as an attachment if it exceeds 20,000 characters)

**Reviewer #1:** The article being reviewed presents solid and well-structured research, with effective use of environmental data and isotopic analysis to assess changes in coastal nitrogen sources. The analysis covers a long period (1989–2023), allowing for the identification of possible long-term trends. However, some critical issues were noted regarding the description and clarity of the materials and methods section. Although the key concepts and main tools are described, essential details are missing, which may hinder the study’s reproducibility.

**We thank the reviewer for her/his comments that help to improve the manuscript. In the new version, we have added the required clarifications on the methodology, including the access to all raw isotopic data.**

Several key issues were identified, including:
1. Lack of details on sampling and sample processing protocols.
o Rows 138–142: It is not clearly specified whether previous studies have used Bifurcaria bifurcata as a biomonitor and whether it could replace Fucus in this role.

**To our knowledge, natural abundance of stable nitrogen isotopes in *Bifurcaria bifurcata* was never used to trace nitrogen sources as biomonitor. However, other studies have considered this species as a potential food source for littoral consumers (e.g. [54]). The rationale for using *Bifurcaria* is the same as for *Fucus*: a species with ample distribution in the region, receiving nitrogen inputs from several potential sources (upwelling, runoff, atmospheric inputs, wastewaters, etc.) and important as resource for littoral food webs.**

**We have modified the text to clarify this point:**

**“To ensure maximum geographical and temporal coverage across the selected zones (Fig 1), several species of *Fucus* were considered (*F. vesiculosus*, *F. spiralis*, and *F. serratus*). When these species were not present (13% of cases) *Bifurcaria bifurcata* was used. The latter was a warm water species gradually replacing *Fucus* spp. in the southern Bay of Biscay [53] and contributes to nitrogen transfer in littoral food webs [54].”**

o Row 151 mentions sampling a portion of the mussels adductor muscle but does not specify the exact quantity. Does this refer to a specific weight (grams) or length (centimetres), or is the quantity irrelevant?

**The exact quantity of muscle analyzed for stable isotopes is not relevant here. Natural abundance of stable isotopes is referred as delta units in ‰, relative to the isotopic composition of atmospheric nitrogen [58]. All isotopic determinations in this study used optimal sample amounts to ensure the indicated precision in δ^15^N (< 0.03 ‰). The amount of sample introduced in the mass spectrometer depends on the absolute amount of nitrogen present in the sample. Therefore, samples of mussel muscle need to be smaller than those of macroalgae, as the former contain mainly protein while macroalgal tissues are composed mainly of carbohydrates. In addition, each particular instrument configuration may have specific requirements of sample size, thus, the settings applied in this study may not be appropriate in other cases. Stable isotope laboratories routinely examine optimal sample size for different matrices.**

2. Insufficient description of sample preservation. What measures were taken to prevent biological degradation after sampling?

**Sample preservation was ensured by removing water to prevent microbial degradation of organic matter. In this case, the degradation would lead to the enrichment in heavy nitrogen isotopes (^15^N), as the lighter isotope (^14^N) would be preferentially released. Besides, the isotopic determinations require a dried sample. While freezing is generally employed for sample preservation, the risk of accidental defrosting, particularly during long term storage, is high. Oven-drying (45-50°C) is the most common procedure used to prepare samples for stable isotope analysis but freeze-drying has the advantage to preserve volatile compounds (e.g. fatty acids) that are lost at high temperature. In our case, as we focus on nitrogen compounds that are mainly contained in proteins, the drying method is not relevant. In contrast, a long storage time before the analysis would require additional tests to ensure that sample degradation (i.e. ^15^N enrichment) did not occurred. However, in our study we analyzed the samples within six months after sampling and storage in dry containers with desiccant. Further details are provided in previous articles using part of the data [45, 46, 54].**

**We have added a clarification in the methods section: “Samples were stored in dry containers for up to 6 months before analysis”.**

3. Incomplete documentation of laboratory instruments and procedures.
o Row 121: It is recommended to specify exactly what the CTD is, including its model and parameters.

**The CTD model is now indicated: “Profiles of temperature and salinity were obtained with a CTD (Seabird SBE25)”**

o Rows 151–153: The rationale behind the choice of drying or freeze-drying some samples is unclear. Was an existing protocol followed? If so, please specify which one. Otherwise, all steps leading to the preparation of samples for isotopic analysis should be described.

**See the response to point 2 above.**

o Rows 142–143: If nitrogen isotopic measurements were extracted from the Environmental Specimen Bank of Galicia, it would be appropriate to specify exactly what type of information was selected. Were filters or other selection criteria used? Additionally, providing a link to this database would enhance transparency.

**All isotopic data used in this study are compiled in the PANGAEA repository as indicated at the end of the “Stable isotopes in intertidal sentinels” section: “All isotopic measurements considered in this study (including the amino acid δ^15^N values in mussels) are available at the PANGAEA repository (http://www.pangaea.de, [61, 62]).”**

**The selection criteria for values obtained from the Environmental Specimen Bank of Galicia was the same as for other samples and was already specified in the text: “Both algae and mussels were collected at mid to low intertidal levels during low tide at wave-exposed locations outside rías and estuaries, avoiding those places near waste-water treatment plants and large urban settlements, thus maximizing the probability of detecting the influence of marine nitrogen sources [45].”**

**There is no URL link for the Environmental Specimen Bank of Galicia but we have provided a relevant reference where further details on sampling and analysis can be found [46].**

o Rows 149–150: It is not specified whether previous studies have observed correlations between isotopic signatures and shell size.

**We agree. We now say: “The shell length of the mussels (as a proxy for age) was measured to investigate potential ontogenetic changes of isotopic composition [56].”**

It is recommended that the materials and methods section be rewritten in a more comprehensive manner, as the current version lacks essential information needed to ensure reproducibility. If including all details in the main text would be overly cumbersome, they could be provided in tables, appendices, or supplementary materials, but they must be made available.

**As suggested by the reviewer, we have added clarifications on all subsections of the Materials and Methods section and provided additional references where further methodological details can be found. We note that there were several specific studies based on part of the isotopic data compiled for the present study (see our response to the point 4 raised by the editor).**

The quality of the figures is generally good, but some aspects could be improved to enhance readability and accessibility:

• Figure 1: Although latitude and longitude references are provided, the figure lacks a metric scale, which would aid in understanding the depicted distances. It is also suggested to use a colour other than black to indicate algae sampling areas, as this is difficult to distinguish from the land outline. Additionally, using a thicker symbol for Mytilus would improve visibility.

**We have modified Fig 1 to include a metric scale and revised the color palette to enhance visibility (see our response to the following point).**

• To enhance accessibility, it is recommended to use a colour-blind-friendly palette, such as the one proposed by Wong, B. (2011). Points of View: Colour Blindness. Nat Methods, 8(441). <https://doi.org/10.1038/nmeth.1618>

**Color figures were redrawn using the color blind-friendly Viridis palette (https://waldyrious.net/viridis-palette-generator/)**

It is advised that the authors carefully check the formatting of the article. For example:
• Row 738: 15N should be formatted as a superscript.

**The manuscript has been thoroughly revised to comply with the formatting requirements.**

**Reference 72 was corrected: “72. Mariotti A (1983) Atmospheric nitrogen is a reliable standard for natural ^15^N abundance measurements. Nature 303(5919): 685-687. https://doi.org/10.1038/303685a0”**

• Citations: References [19], [20], and [21] appear before [16], while [17] and [18] are missing. It is possible that in line 48, the citation indicated as [15, 19] should actually be [15–19].

**New references were added and the numbering of all references was revised**

6. PLOS authors have the option to publish the peer review history of their article ([what does this mean?](https://journals.plos.org/plosone/s/editorial-and-peer-review-process#loc-peer-review-history)). If published, this will include your full peer review and any attached files.

If you choose “no”, your identity will remain anonymous but your review may still be made public.

**Do you want your identity to be public for this peer review?** For information about this choice, including consent withdrawal, please see our [Privacy Policy](https://www.plos.org/privacy-policy).

Reviewer #1: No

**Additional references:**

**Arístegui J, Alvarez-Salgado XA, Barton ED, Figueiras FG, Hernández-León S et al. (2006) Chapter 23. Oceanography and fisheries of the Canary Current/Iberian region of the Eastern North Atlantic (18a, E). In: Robinson AR, Brink K, editors. The Global Coastal Ocean: Interdisciplinary Regional Studies and Syntheses. Boston: Harvard University Press. pp. 877-931.**

**Bode A, Varela M, Prego R (2011) Continental and marine sources of organic matter and nitrogen for rías of northern Galicia (Spain). Marine Ecology Progress Series 437: 13-26.** [**https://doi.org/10.3354/meps318089**](https://doi.org/10.3354/meps318089)**.**

**Casado-Amezúa P, Araújo R, Bárbara I, Bermejo R, Borja Á et al. (2019) Distributional shifts of canopy-forming seaweeds from the Atlantic coast of Southern Europe. Biodiversity and Conservation 28(5): 1151-1172.** [**https://doi.org/10.1007/s10531-019-01716-9**](https://doi.org/10.1007/s10531-019-01716-9)**.**

**Figueiras FG, Labarta U, Fernández Reiriz MJ (2002) Coastal upwelling, primary production and mussel growth in the Rías Baixas of Galicia. Hydrobiologia 484: 121-131.** [**https://doi.org/10.1023/A:1021309222459**](https://doi.org/10.1023/A:1021309222459)**.**
